# 2-Hy­droxy­methyl-1,3-dimethyl­imidazolium iodide

**DOI:** 10.1107/S1600536811025700

**Published:** 2011-07-02

**Authors:** Meryem Chelghoum, Mebarek Bahnous, Sofiane Bouacida, Thierry Roisnel, Ali Belfaitah

**Affiliations:** a Laboratoire des Produits Naturels d’Origine Végétale et de Synthèse Organique, PHYSYNOR, Université Mentouri-Constantine, 25000 Constantine, Algeria; b Unité de Recherche de Chimie de l’Environnement et Moléculaire Structurale, CHEMS, Université Mentouri-Constantine 25000, Algeria; c Centre de Difractométrie X, UMR 6226 CNRS Unité Sciences Chimiques de Rennes, Université de Rennes I, 263 Avenue du Général Leclerc, 35042 Rennes, France

## Abstract

The crystal packing of the title compound, C_6_H_11_N_2_O^+^·I^−^, can be described as inter­calated layers lying parallel to (010), with the iodide ions located between the cations. A weak intra­molecular C—H⋯O hydrogen bond occurs within the cation. In the crystal, inter­molecular O—H⋯I hydrogen bonds result in the formation of a three-dimensional network and reinforce the cohesion of the ionic structure.

## Related literature

For related ionic liquids, see: Welton (1999 ▶); Kubisa (2004 ▶); Corma & Garcia (2003 ▶); Sheldon (2001 ▶); Wasserscheid & Kerm (2000 ▶). For synthetic appilications of ionic liquids, see: Varma & Namboodiri (2001 ▶).
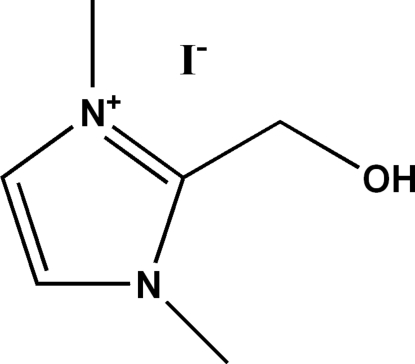


## Experimental

### 

#### Crystal data

C_6_H_11_N_2_O^+^·I^−^
*M* *_r_* = 254.07
Monoclinic, 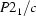

*a* = 7.3428 (3) Å
*b* = 7.2186 (3) Å
*c* = 16.8870 (8) Å
β = 93.093 (2)°
*V* = 893.79 (7) Å^3^
*Z* = 4
Mo *K*α radiation
μ = 3.53 mm^−1^
*T* = 150 K
0.3 × 0.13 × 0.01 mm

#### Data collection

Bruker APEXII diffractometer
Absorption correction: multi-scan (*SADABS*; Sheldrick, 2002 ▶) *T*_min_ = 0.718, *T*_max_ = 0.965
4200 measured reflections
2035 independent reflections
1463 reflections with *I* > 2σ(*I*)
*R*_int_ = 0.019

#### Refinement

*R*[*F* ^2^ > 2σ(*F* ^2^)] = 0.021
*wR*(*F* ^2^) = 0.063
*S* = 1.03
2035 reflections
94 parameters
H-atom parameters constrained
Δρ_max_ = 0.66 e Å^−3^
Δρ_min_ = −0.54 e Å^−3^

### 

Data collection: *APEX2* (Bruker, 2006 ▶); cell refinement: *SAINT* (Bruker, 2001 ▶); data reduction: *SAINT*; program(s) used to solve structure: *SIR2002* (Burla *et al.*, 2005 ▶); program(s) used to refine structure: *SHELXL97* (Sheldrick, 2008 ▶); molecular graphics: *ORTEP-3 for Windows* (Farrugia, 1997 ▶) and *DIAMOND* (Brandenburg & Berndt, 2001 ▶); software used to prepare material for publication: *WinGX* (Farrugia, 1999 ▶).

## Supplementary Material

Crystal structure: contains datablock(s) global, I. DOI: 10.1107/S1600536811025700/hg5062sup1.cif

Structure factors: contains datablock(s) I. DOI: 10.1107/S1600536811025700/hg5062Isup2.hkl

Supplementary material file. DOI: 10.1107/S1600536811025700/hg5062Isup3.cml

Additional supplementary materials:  crystallographic information; 3D view; checkCIF report

## Figures and Tables

**Table 1:** Hydrogen-bond geometry (Å, °)

*D*—H⋯*A*	*D*—H	H⋯*A*	*D*⋯*A*	*D*—H⋯*A*
O9—H9⋯I1	0.84	2.62	3.4504 (18)	169
C1—H1*A*⋯O9	0.98	2.55	3.230 (4)	126

**Fig. 1.**
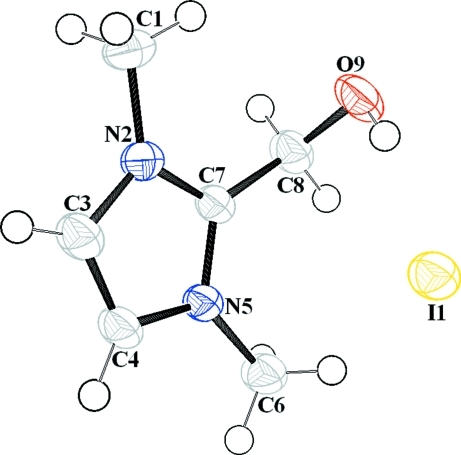
(Farrugia, 1997) the structure of the title compound with the atomic labelling scheme.Displacement are drawn at the 50% probability level.

**Fig. 2.**
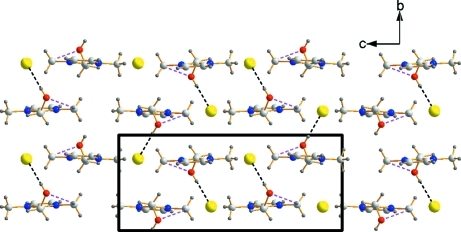
(Brandenburg & Berndt, 2001) A diagram of the layered crystal packing of (I) viewed down the a axis.

**Fig. 3.**
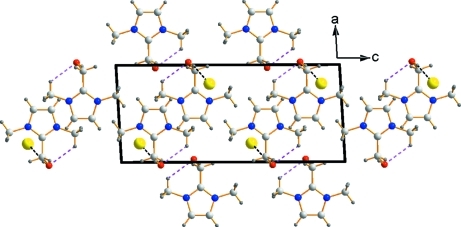
(Brandenburg & Berndt, 2001)Part of crystal packing of (I) showing hydrogen bond [C—H···O, O—H···I] as dashed line.

## supplementary crystallographic information

### Comment

The development of cleaner technologies is a major emphasis in green chemistry. Among the several aspects of green chemistry, the reduction/replacement of volatile organic solvents from the reaction medium is of utmost importance. The use of a large excess of conventional volatile solvents required to conduct a chemical reaction creates ecological and economic concerns. The search for a nonvolatile and recyclable alternative is thus holding a key role in this field of research. The use of fused organic salts, consisting of ions, is now emerging as a possible alternative. A proper choice of cations and anions is required to achieve ionic salts that are liquids at room temperature and are appropriately termed room temperature ionic liquids (RTILs) (Welton, 1999; Kubisa 2004; Corma & Garcia 2003; Sheldon, 2001; Wasserscheid & Kerm, 2000). The ionic liquids based on 1,3-dialkylimidazolium are becoming more important for several synthetic applications (Varma & Namboodiri 2001).

In this work, we report synthesis and the structure determination of an ionic compound obtained from the quaternization reaction of 1-methyl-2-hydroxymethylimidazole using methyl iodide (I).

The molecular geometry and the atom-numbering scheme of (I) are shown in Fig. 1. The asymetric unit of title molecule, C_6_H_11_N_2_O^+^, I^-^, contains a 2-hydroxymethyl-1,3-dimethylimidazolium cation and iodide anion.

The crystal packing can be described as intercalated layers parallel to the (010) plane, wich iodide ions are located between cations (Fig. 2). It is stabilized by weak intra and intermolecular hydrogen bonds [O—H···I and C—H···O] (Fig. 3). These interaction bonds link the molecules within the layers and also link the layers together, forming a three dimensional network and reinforcing the cohesion of the ionic structure. Hydrogen-bonding parameters are listed in table 1.

### Experimental

The title compound I was synthesized by treating 1eq of (1-methyl-1*H*-imidazol-2-yl)methanol by 3 eq of methyl iodide in refluxing THF during two days. The solid is filtered off and washed with boiling THF. Suitable crystals of I were obtained by crystallization from a CH_3_CN/THF solution.

### Refinement

All non-H atoms were refined with anisotropic atomic displacement parameters. All H atoms were localized on Fourier maps but introduced in calculated positions and treated as riding on their parent C or O atom. (with C—H = 0.95Å 0.98Å 0.99 Å, O—H = 84 Å and *U*_iso_(H) =1.2 or 1.5(carrier atom)).

### Crystal data

**Table:**

C_6_H_11_N_2_O^+^·I^−^	*F*(000) = 488
*M**_r_* = 254.07	*D*_x_ = 1.888 Mg m^−^^3^
Monoclinic, *P*2_1_/*c*	Mo *K*α radiation, λ = 0.71073 Å
Hall symbol: -P 2ybc	Cell parameters from 1935 reflections
*a* = 7.3428 (3) Å	θ = 2.4–27.4°
*b* = 7.2186 (3) Å	µ = 3.53 mm^−^^1^
*c* = 16.8870 (8) Å	*T* = 150 K
β = 93.093 (2)°	Prism, colourless
*V* = 893.79 (7) Å^3^	0.3 × 0.13 × 0.01 mm
*Z* = 4	

### Data collection

**Table:**

Bruker APEXII diffractometer	1463 reflections with *I* > 2σ(*I*)
graphite	*R*_int_ = 0.019
CCD rotation images, thin slices scans	θ_max_ = 27.4°, θ_min_ = 3.6°
Absorption correction: multi-scan (*SADABS*; Sheldrick, 2002)	*h* = −5→9
*T*_min_ = 0.718, *T*_max_ = 0.965	*k* = −6→9
4200 measured reflections	*l* = −21→21
2035 independent reflections	

### Refinement

**Table:**

Refinement on *F*^2^	Primary atom site location: structure-invariant direct methods
Least-squares matrix: full	Secondary atom site location: difference Fourier map
*R*[*F*^2^ > 2σ(*F*^2^)] = 0.021	Hydrogen site location: inferred from neighbouring sites
*wR*(*F*^2^) = 0.063	H-atom parameters constrained
*S* = 1.03	*w* = 1/[σ^2^(*F*_o_^2^) + (0.0289*P*)^2^ + 0.3175*P*] where *P* = (*F*_o_^2^ + 2*F*_c_^2^)/3
2035 reflections	(Δ/σ)_max_ = 0.001
94 parameters	Δρ_max_ = 0.66 e Å^−^^3^
0 restraints	Δρ_min_ = −0.54 e Å^−^^3^

### Special details

**Table:**

Geometry. All e.s.d.'s (except the e.s.d. in the dihedral angle between two l.s. planes) are estimated using the full covariance matrix. The cell e.s.d.'s are taken into account individually in the estimation of e.s.d.'s in distances, angles and torsion angles; correlations between e.s.d.'s in cell parameters are only used when they are defined by crystal symmetry. An approximate (isotropic) treatment of cell e.s.d.'s is used for estimating e.s.d.'s involving l.s. planes.
Refinement. Refinement of *F*^2^ against ALL reflections. The weighted *R*-factor *wR* and goodness of fit *S* are based on *F*^2^, conventional *R*-factors *R* are based on *F*, with *F* set to zero for negative *F*^2^. The threshold expression of *F*^2^ > σ(*F*^2^) is used only for calculating *R*-factors(gt) *etc*. and is not relevant to the choice of reflections for refinement. *R*-factors based on *F*^2^ are statistically about twice as large as those based on *F*, and *R*- factors based on ALL data will be even larger.

### Fractional atomic coordinates and isotropic or equivalent isotropic displacement parameters (Å^2^)

**Table:**

	*x*	*y*	*z*	*U*_iso_*/*U*_eq_	
I1	0.80624 (2)	0.74882 (2)	0.413580 (10)	0.02430 (8)	
C1	0.6996 (5)	0.2724 (3)	0.20049 (17)	0.0284 (6)	
H1A	0.8326	0.2864	0.2033	0.043*	
H1B	0.6438	0.3775	0.1716	0.043*	
H1C	0.6663	0.157	0.1728	0.043*	
N2	0.6343 (3)	0.2668 (2)	0.28084 (14)	0.0204 (5)	
C3	0.4551 (4)	0.2855 (3)	0.29967 (18)	0.0251 (6)	
H3	0.3528	0.3011	0.2634	0.03*	
C4	0.4528 (4)	0.2773 (3)	0.37988 (17)	0.0232 (6)	
H4	0.3482	0.2865	0.4104	0.028*	
N5	0.6304 (3)	0.2533 (2)	0.40871 (13)	0.0195 (5)	
C6	0.6882 (4)	0.2431 (3)	0.49319 (18)	0.0271 (6)	
H6A	0.7534	0.1265	0.5038	0.041*	
H6B	0.5807	0.2483	0.5251	0.041*	
H6C	0.7689	0.3475	0.5071	0.041*	
C7	0.7404 (4)	0.2472 (3)	0.34775 (16)	0.0192 (5)	
C8	0.9437 (4)	0.2288 (3)	0.35238 (19)	0.0254 (6)	
H8A	0.9867	0.2055	0.4081	0.03*	
H8B	0.9789	0.1212	0.3202	0.03*	
O9	1.0295 (3)	0.3905 (2)	0.32453 (12)	0.0305 (4)	
H9	0.9902	0.4838	0.348	0.046*	

### Atomic displacement parameters (Å^2^)

**Table:**

	*U*^11^	*U*^22^	*U*^33^	*U*^12^	*U*^13^	*U*^23^
I1	0.01963 (12)	0.02953 (13)	0.02391 (12)	0.00057 (7)	0.00275 (8)	−0.00027 (6)
C1	0.0296 (16)	0.0386 (16)	0.0173 (14)	−0.0004 (12)	0.0043 (12)	−0.0005 (11)
N2	0.0192 (11)	0.0243 (11)	0.0179 (11)	0.0006 (8)	0.0020 (9)	−0.0003 (8)
C3	0.0170 (14)	0.0308 (15)	0.0277 (15)	0.0020 (11)	0.0027 (11)	0.0000 (11)
C4	0.0147 (13)	0.0310 (14)	0.0242 (14)	0.0011 (10)	0.0029 (11)	0.0007 (10)
N5	0.0154 (11)	0.0256 (11)	0.0176 (11)	0.0004 (8)	0.0017 (9)	−0.0005 (8)
C6	0.0209 (14)	0.0408 (17)	0.0196 (14)	0.0001 (11)	0.0011 (11)	0.0017 (10)
C7	0.0161 (12)	0.0217 (13)	0.0200 (13)	−0.0007 (10)	0.0026 (10)	0.0003 (9)
C8	0.0172 (14)	0.0315 (15)	0.0276 (15)	0.0006 (11)	0.0026 (12)	0.0005 (11)
O9	0.0217 (10)	0.0301 (10)	0.0410 (11)	−0.0052 (9)	0.0133 (8)	−0.0041 (9)

### Geometric parameters (Å, °)

**Table:**

C1—N2	1.464 (4)	N5—C7	1.343 (3)
C1—H1A	0.98	N5—C6	1.468 (4)
C1—H1B	0.98	C6—H6A	0.98
C1—H1C	0.98	C6—H6B	0.98
N2—C7	1.345 (3)	C6—H6C	0.98
N2—C3	1.376 (4)	C7—C8	1.497 (4)
C3—C4	1.357 (4)	C8—O9	1.419 (3)
C3—H3	0.95	C8—H8A	0.99
C4—N5	1.379 (4)	C8—H8B	0.99
C4—H4	0.95	O9—H9	0.84
			
N2—C1—H1A	109.5	C4—N5—C6	124.6 (2)
N2—C1—H1B	109.5	N5—C6—H6A	109.5
H1A—C1—H1B	109.5	N5—C6—H6B	109.5
N2—C1—H1C	109.5	H6A—C6—H6B	109.5
H1A—C1—H1C	109.5	N5—C6—H6C	109.5
H1B—C1—H1C	109.5	H6A—C6—H6C	109.5
C7—N2—C3	109.5 (2)	H6B—C6—H6C	109.5
C7—N2—C1	125.3 (3)	N5—C7—N2	107.2 (3)
C3—N2—C1	125.2 (3)	N5—C7—C8	127.0 (3)
C4—C3—N2	106.9 (3)	N2—C7—C8	125.7 (3)
C4—C3—H3	126.6	O9—C8—C7	111.6 (2)
N2—C3—H3	126.6	O9—C8—H8A	109.3
C3—C4—N5	107.2 (3)	C7—C8—H8A	109.3
C3—C4—H4	126.4	O9—C8—H8B	109.3
N5—C4—H4	126.4	C7—C8—H8B	109.3
C7—N5—C4	109.3 (2)	H8A—C8—H8B	108
C7—N5—C6	126.1 (3)	C8—O9—H9	109.5
			
C7—N2—C3—C4	0.1 (3)	C6—N5—C7—C8	0.1 (3)
C1—N2—C3—C4	−178.4 (2)	C3—N2—C7—N5	0.0 (2)
N2—C3—C4—N5	−0.2 (3)	C1—N2—C7—N5	178.48 (18)
C3—C4—N5—C7	0.2 (2)	C3—N2—C7—C8	−178.1 (2)
C3—C4—N5—C6	178.15 (19)	C1—N2—C7—C8	0.3 (3)
C4—N5—C7—N2	−0.1 (2)	N5—C7—C8—O9	−113.9 (3)
C6—N5—C7—N2	−178.06 (18)	N2—C7—C8—O9	63.8 (3)
C4—N5—C7—C8	178.0 (2)		

### Hydrogen-bond geometry (Å, °)

**Table:**

*D*—H···*A*	*D*—H	H···*A*	*D*···*A*	*D*—H···*A*
O9—H9···I1	0.84	2.62	3.4504 (18)	169
C1—H1A···O9	0.98	2.55	3.230 (4)	126
